# The landscape of hervRNAs transcribed from human endogenous retroviruses across human body sites

**DOI:** 10.1186/s13059-022-02804-w

**Published:** 2022-11-03

**Authors:** Jianqi She, Minghao Du, Zhanzhan Xu, Yueqi Jin, Yu Li, Daoning Zhang, Changyu Tao, Jian Chen, Jiadong Wang, Ence Yang

**Affiliations:** 1 Department of Microbiology & Infectious Disease Center, School of Basic Medical Sciences, Peking University Health Science Center, Key Laboratory for Neuroscience, Ministry of Education/National Health Commission of China, NHC Key Laboratory of Medical Immunology (Peking University), Beijing, 100191 China; 2 Department of Medical Bioinformatics, School of Basic Medical Sciences, Peking University Health Science Center, Beijing, 100191 China; 3 Department of Radiation Medicine, School of Basic Medical Sciences, Peking University Health Science Center, Beijing, 100191 China; 4 Chinese Institute for Brain Research, Beijing, 102206 China; 5 Peking University First Hospital, Beijing, 100034 China; 6 Department of Human Anatomy, Histology & Embryology, School of Basic Medical Sciences, Peking University Health Science Center, Beijing, 100191 China; 7 Taizhou Medical New & Hi-tech Industrial Development Zone, Jiangsu, 225326 China

## Abstract

**Background:**

Human endogenous retroviruses (HERVs), the remnants of ancient retroviruses, account for 8% of the human genome, but most have lost their transcriptional abilities under physiological conditions. However, mounting evidence shows that several expressed HERVs do exert biological functions. Here, we systematically characterize physiologically expressed HERVs and examine whether they may give insight into the molecular fundamentals of human development and disease.

**Results:**

We systematically identify 13,889 expressed HERVs across normal body sites and demonstrate that they are expressed in body site-specific patterns and also by sex, ethnicity, and age. Analyzing *cis*-ERV-related quantitative trait loci, we find that 5435 hervRNAs are regulated by genetic variants. Combining this with a genome-wide association study, we elucidate that the dysregulation of expressed HERVs might be associated with various complex diseases, particularly neurodegenerative and psychiatric diseases. We further find that physiologically activated hervRNAs are associated with histone modifications rather than DNA demethylation.

**Conclusions:**

Our results present a locus-specific landscape of physiologically expressed hervRNAs, which represent a hidden layer of genetic architecture in development and disease.

**Supplementary Information:**

The online version contains supplementary material available at 10.1186/s13059-022-02804-w.

## Background

Human endogenous retroviruses (HERVs), derived from ancient retroviral integration into the germline, are a class of transposable elements that constitute approximately 8% of the human genome [1, 2]. Due to cumulative mutations throughout evolution and epigenetic inhibition by the host, most HERVs have lost their transcriptional ability under physiological conditions [3]. However, accumulating evidence highlights the novel and indispensable functionalities of HERV-derived elements in human development [4]. For example, syncytin-1 (encoded by *ERVW-1*) and syncytin-2 (encoded by *ERVFRD-1*) play critical roles in syncytialization during placental morphogenesis [5, 6]; transcriptionally active HERV-H elements are able to affect gene regulatory programs and even create novel topologically associating domains (TADs) [7, 8]. In addition, a few multi-tissue studies based on microarray analysis suggest that HERVs are abundantly expressed in normal tissues [9, 10]. Thus, systemic characterization of expressed HERVs in normal tissues will extend our understanding of transcriptional diversity and complexity during human development.

Given their repetitive nature, expressed HERVs are usually detected at the family level, i.e., by clustering multiple HERV elements with consensus sequences into a unit to simplify identification. However, such family-level approaches are unable to analyze locus-specific regulatory mechanisms and explain whether the observations represent the general characteristics of all HERV elements within a family or not [11]. To detect locus-specific HERVs, a straightforward approach is to discard multiple mapping reads by setting stringent criteria, which also reduces the number of expressed HERVs identified [12, 13]. Alternatively, approaches such as TEtranscripts and Telescope implement heuristic or statistical models to estimate the assignment of multiple mapping reads, but these approaches count signals at hundred thousands of individual HERV elements rather than actual transcripts, and thus may aggregate noise with large numbers of false-positive candidates [14–16]. On the contrary, an assembly-based strategy provides an opportunity to achieve actual transcripts from HERV elements, which may lead to novel insights into physiologically expressed HERVs [17–19].

Here, we applied a genome-guided de novo assembly strategy in the locus-specific identification of expressed HERVs. By analyzing 9466 RNA-seq samples from the Genotype-Tissue Expression (GTEx) Project, we identified 13,889 expressed HERVs across 42 human body sites and revealed body site-specific expression patterns as well as biology (sex, ethnicity, and age)-associated patterns. By *cis*-ERV-related quantitative trait loci (*cis*-ervQTLs) analysis, we elucidated that the expression of HERV is regulated by genetic variants. Combining with genome-wide association study (GWAS) variants, we revealed that the dysregulation of expressed HERVs might be associated with various complex diseases. With ENTEx data generated by the Encyclopedia of DNA Elements (ENCODE) Project [20, 21] and the GTEx Project, we implemented the potential epigenetic regulation of HERV expression under physiological conditions. Together, our findings will further our understanding of the roles of physiological expressed HERVs in the genetic architectures of complex traits and diseases.

## Results

### Detection of expressed HERVs

**Fig. 1:**
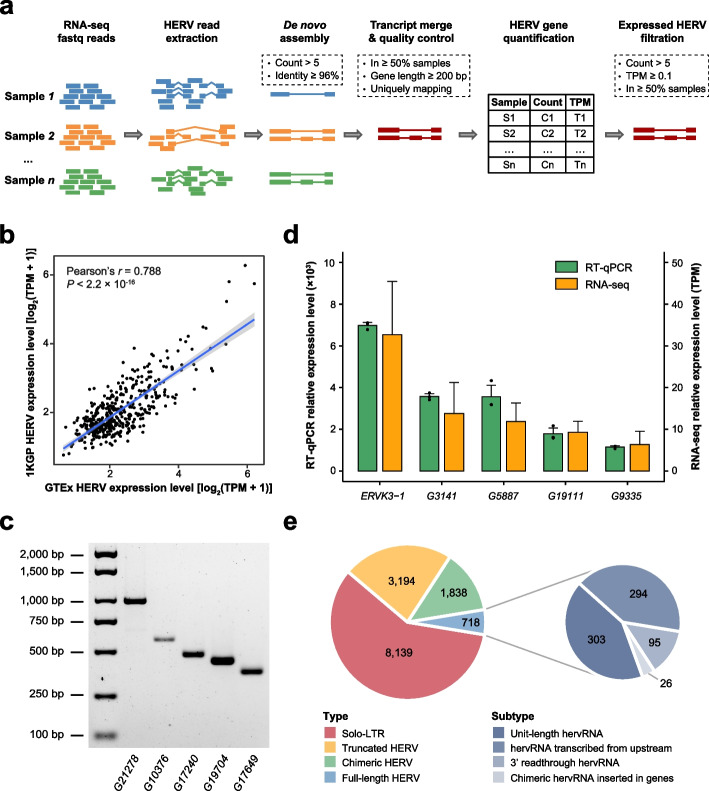
Detection of expressed HERVs. **a** Pipeline designed to identify and quantify expressed HERVs from RNA-seq data. **b** Pearson correlation of the expression levels of HERVs between GTEx and 1KGP. The shaded area around the regression line represents the 95% confidence interval. **c** Gel electrophoresis image of the target RT-PCR HERV product bands. G21278: *Fibroblasts_HERV_G21278*; G10376: *Fibroblasts_HERV_G10376*; G17240: *Fibroblasts_HERV_G17240*; G19704: *Fibroblasts_HERV_G19704*; and G17649: *Fibroblasts_HERV_G17649*. **d** Comparison of the expression levels of HERVs between RT-qPCR and RNA-seq results, shown as the mean ± standard deviation (SD). **e** Classification of expressed HERVs based on the HERVd database

Based on an assembly-based strategy, we improved a pipeline with strict criteria to de novo identified high-quality expressed HERV elements from RNA-seq data (Fig. 1a, see details in the “Methods” section). We first applied our pipeline to the 1000 Genomes Project (1KGP) [22, 23] and identified 473 expressed HERVs, 93% of which were also detected in data for EBV-transformed lymphocytes from the GTEx Project. For these commonly expressed HERVs, the expression levels were significantly consistent (Fig. 1b). Then, we identified 1110 expressed HERVs in human skin fibroblasts (HSFs) from the GTEx Project and randomly selected five expressed HERVs for experimental validation. Within in-house cultured HSF cell lines, full-length sequences of all target HERVs were verified by reverse transcription PCR (RT-PCR) and Sanger sequencing (Fig. 1c, Additional file 1: Fig. S1, Additional file 2: Table S1). We also randomly selected another five HSF-expressed HERVs for quantitative reverse transcription PCR (RT-qPCR), which exhibited high consistency with the detection by our pipeline (Fig. 1d, Additional file 2: Table S1). Together, the results support that our pipeline is sufficient for the identification and quantification of expressed HERVs.

By analyzing 9466 RNA-seq datasets from 686 individuals included in the GTEx Project, we systemically identified a total of 13,889 locus-specific expressed HERVs (hervRNAs or human ervRNAs) across 42 human body sites (Additional file 2: Table S2). Based on location, these hervRNAs were categorized into 6681 antisense hervRNAs, 4471 intergenic hervRNAs, and 2737 inserted hervRNAs. With annotation from HERVd database, the origin of these hervRNAs were classified into four groups, including solo-LTRs (*n* = 8,139), truncated HERVs (*n* = 3,194), chimeric HERVs (concatenating neighboring HERV elements, *n* = 1,838), and full-length HERVs (*n* = 718; Fig. 1e and Additional file 2: Table S2). Specially, coding potential was detected in 110 hervRNAs, deriving from 28 truncated HERVs, 39 chimeric HERVs, and 43 full-length HERVs (Additional file 2: Table S2). Following the categories in a recent work [24], the full-length HERVs were further classified into 303 unit-length hervRNAs with intact structure, 294 of hervRNAs transcribed from upstream [transcription start site (TSS) is located at the upstream of HERV element], 95 of 3′ readthrough hervRNAs, and 26 of chimeric hervRNAs inserted in the transcripts from host genes (Fig. 1e and Additional file 2: Table S2).

### Global atlas of hervRNAs across human body sites

**Fig. 2:**
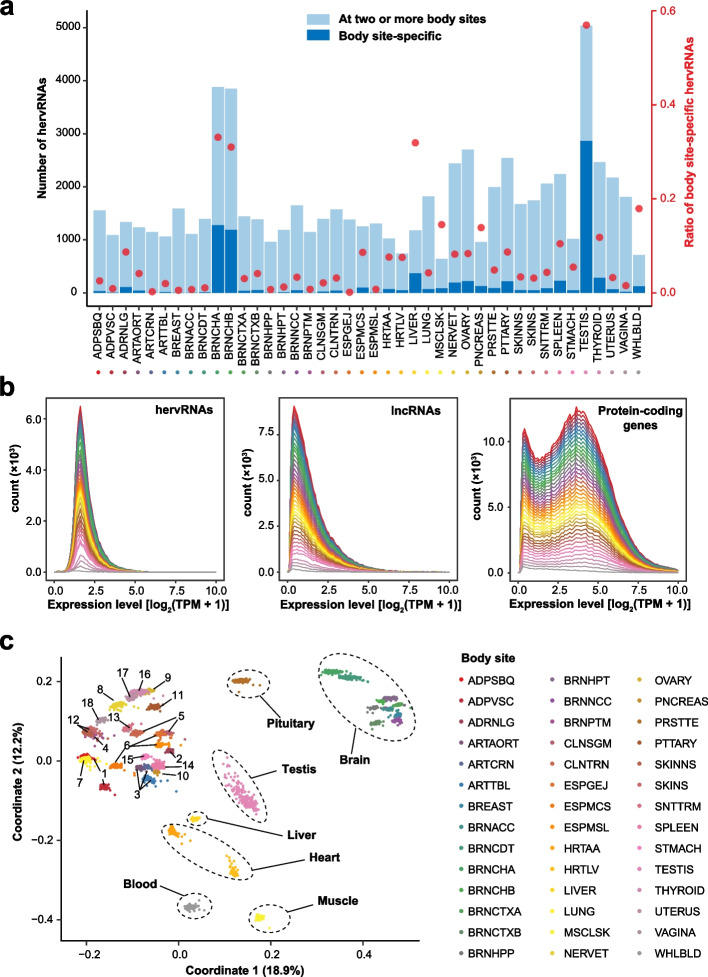
hervRNAs across body sites in GTEx. **a** Distribution of hervRNAs across 42 body sites. Blue stacked bars indicate the number of hervRNAs (left *y*-axis), and red dots indicate the ratio of body site-specific hervRNAs at each body site (right *y*-axis). The colors assigned to each body site are indicated on the *x*-axis. **b** Frequency polygons of the expression levels of hervRNAs (left), lncRNAs (middle), and protein-coding genes (right) across body sites. See **a** for the legend of body site-related colors. **c** Sample similarity based on hervRNA profiles by multidimensional scaling. Tissues in the upper left corner are as follows: 1: adipose tissue; 2: adrenal gland; 3: blood vessel; 4: breast; 5: colon; 6: esophagus; 7: lung; 8: nerve; 9: ovary; 10: pancreas; 11: prostate; 12: skin; 13: small intestine; 14: spleen; 15: stomach; 16: thyroid; 17: uterus; 18: vagina

Across different body sites, the counts of hervRNAs ranged from 640 in the muscle skeletal to 5035 in the testis (Fig. 2a, Additional file 2: Table S3). Strikingly, nearly four thousand hervRNAs were detected in the cerebellum (brain-cerebellum: *n* = 3848; brain-cerebellar hemisphere: *n* = 3,881), which was higher than the numbers at other body sites (amount: 640–2699) except for the testis (*n* = 5035). Across body sites, hervRNAs account for 0.19–1.91% of poly(A)-tailed transcripts, which was lower than the percentages of lncRNAs (0.91–5.97%) and protein-coding genes (45.89–95.98%). However, the median expression levels of hervRNAs varied from 1.56 to 3.16 TPM across body sites, which were obviously higher values than those of lncRNAs (median: 0.72–1.93 TPM; Fig. 2b). The expression profiles of hervRNAs accurately recapitulated both different body site types and tissue types, especially for brain subregions, which were differentiated more clearly than on the basis of lncRNAs and/or protein-coding genes [25] (Fig. 2c).

More than half of the hervRNAs (7566/13,889) were expressed in a body site-specific manner, especially in the testis (*n* = 2867) and cerebellum (*n* = 1503) (Fig. 2a and Additional file 2: Table S3). Interestingly, 31.9% of the liver hervRNAs were liver-specifically expressed, although only 1178 hervRNAs were identified in the liver. Compared with other body sites, testis-specific hervRNAs were significantly enriched in the LTR12C family, which was highly expressed during early spermatogenic stages [26] (*P* < 2.2 × 10^−16^). Among the hervRNAs expressed in multiple body sites, 66.2% (4106/6196) were differentially expressed between at least two body sites (Additional file 1: Fig. S2). Notably, among the 4302 cerebellum hervRNAs, 1503 were cerebellum specific, and 1308 were expressed at higher levels than at least another body site. In particular, 351 hervRNAs were preferentially expressed (i.e., expressed at significantly higher levels than at any other body site) in the cerebellum.

### Potential function of generally ubiquitously expressed hervRNAs

**Fig. 3:**
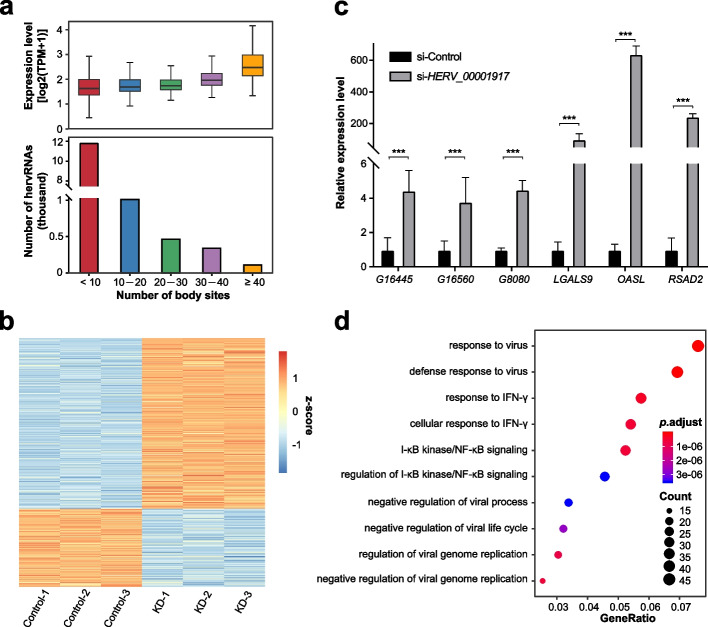
Generally ubiquitously expressed hervRNAs. **a** The number and expression levels of expressed hervRNAs in < 10 body sites, 10–20 body sites, 20–30 body sites, 30–40 body sites, and ≥ 40 body sites. The center line indicates the median, the limits are the interquartile range (IQR), and the whiskers represent 1.5× the IQR. **b** Differentially expressed genes in the *HERV_00001917*-relative module. KD, knockdown. **c** Relative expression of differentially expressed genes and HERVs measured by RT-qPCR in control and *HERV_00001917* siRNA-treated HSFs. Values were normalized to GAPDH and shown as the mean ± SD. G16445: *Fibroblasts_HERV_G16445*; G16560: *Fibroblasts_HERV_G16560*; G8080: *Fibroblasts_HERV_G8080*. ***P < 0.001. **d** Gene Ontology enrichment of differentially expressed genes in the *HERV_00001917*-relative module

We detected 127 generally ubiquitously expressed hervRNAs (in ≥ 40 body sites). As expected, the expression level of these hervRNAs was significantly higher than that of the other hervRNAs (Wilcoxon rank-sum test, false discovery rate (FDR) < 0.05, Fig. 3a). To explore the potential function of these generally ubiquitously expressed hervRNAs, we constructed weighted gene correlation network in HSFs and knocked down *HERV_00001917*, the most highly expressed in the largest module (Additional file 2: Table S4). Strikingly, 2808 differentially expressed genes were detected by RNA sequencing between *HERV_00001917*-knockdown and siRNA-control HSFs. The differentially expressed genes were significantly enriched in the *HERV_00001917*-relative module, supporting the causal roles of *HERV_00001917* in the gene regulatory network (*P* < 2.2 × 10^−16^; Fig. 3 b, c, Additional file 2: Table S5). By Gene Ontology enrichment analysis, the differentially expressed genes in the *HERV_00001917*-relative module were enriched in virus response, IFN-γ response, and NF-κB signaling, suggesting the biological function of *HERV_00001917* in regulating host immunity (Fig. 3d).

### Effects of biological factors on hervRNAs

**Fig. 4:**
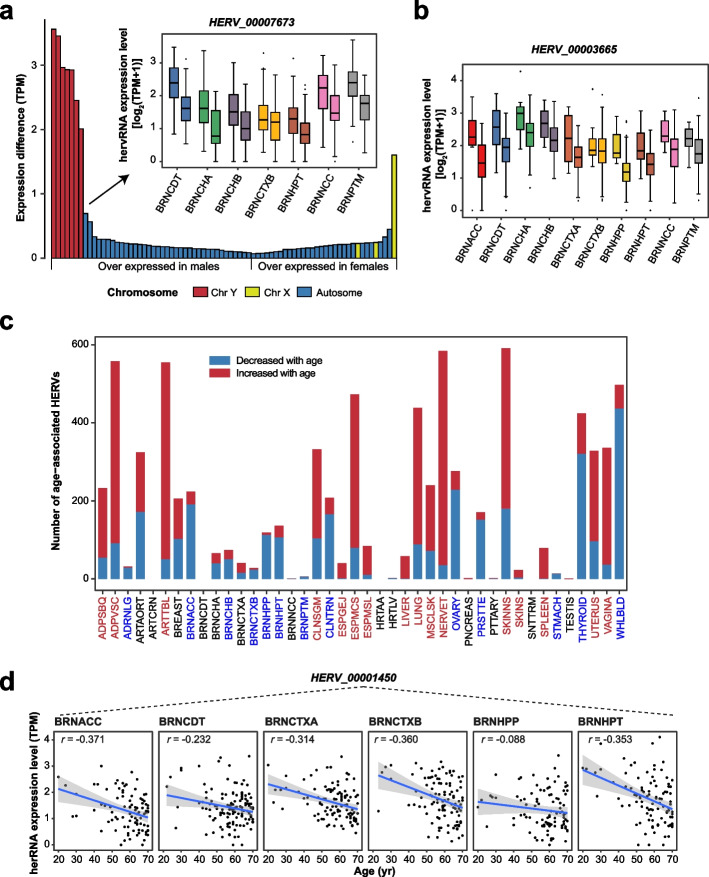
Effects of biological factors on hervRNA expression. **a** Sex differentially expressed hervRNAs. The barplot shows the differentially expressed hervRNAs (FDR < 0.05) ordered by the expression differences between males and females. The boxplot shows *HERV_00007673* gene expression in males (left) and females (right) across 7 body sites. Except for BRNCTXB, the difference between males and females at each body site was significant (*P* < 0.01, Wilcoxon rank-sum test). The center line indicates the median, the limits are the IQR, the whiskers represent 1.5× the IQR, and individual black dots represent outliers. **b ***HERV_00003665* gene expression in African Americans (left) and European Americans (right). Except for BRNCTXB, the difference between males and females at each body site was significant (*P* < 0.01, Wilcoxon rank-sum test). The center line indicates the median, the limits are the IQR, the whiskers represent 1.5× the IQR, and individual black dots represent outliers. **c** The number of age-associated increasing (red) and decreasing (blue) hervRNAs in each body site. The body sites in red indicate enrichment of hervRNA increasing with age while body sites in blue indicate enrichment of hervRNA decreasing with age. **d ***HERV_00001450* gene expression in the brain subregions as a function of age. The shaded area around the regression line represents the 95% confidence interval

To characterize hervRNAs under physiological conditions, we first evaluated the body site-specific and global effects of biological factors including sex, ethnicity, and age. Excluding sex-specific body sites (ovary, prostate, testis, uterus, and vagina), we detected 1095 sex-biased hervRNAs at 37 body sites, most of which were enriched in the breast (*n* = 818; Additional file 2: Table S6). Then, we applied a linear mixed model to these sex-biased hervRNAs to evaluate the global body site effect (more than five body sites). Except for hervRNAs located on the sex chromosome, the most sex-biased hervRNA was *HERV_00007673*, which was brain-specific and was significantly highly expressed in males across 6 brain subregions (Fig. 4a and Additional file 2: Table S7). By contrast, the most sex-biased hervRNA on the autosomes in females is *HERV_00001037*, which was also most biased in breast across body sites.

We also identified 1604 hervRNAs with differential expression between European Americans and African Americans. Interestingly, over 50% of the ethnicity-biased hervRNAs were found in skin tissue (skin-not sun exposed: *n* = 813, skin-sun exposed: *n* = 339; Additional file 2: Table S6), among which 51 belonged to the HERV-K family that has been implicated in melanoma progression [27]. However, all three global ethnicity-biased hervRNAs were preferentially expressed in the brain (Additional file 2: Table S7). Notably, the hervRNA with the most biased expression, *HERV_00003665*, was brain-specific and predominantly expressed in African Americans in almost all brain subregions (Fig. 4b).

We detected 3496 age-associated hervRNAs, which were distributed at almost all body sites except for the coronary artery, brain caudate, heart atrial appendage, and small intestine (Fig. 4c and Additional file 2: Table S6). Interestingly, among the 13 body sites with significant enrichment of age-associated decreasing hervRNAs, six of the sites were within brain tissue, with the brain hippocampus showing especially high enrichment (113/119). A total of 1084 hervRNAs were globally associated with age (Additional file 2: Table S7). Seven of the top 10 age-associated hervRNAs were brain-specific. The hervRNA associated with age showing the greatest decrease was *HERV_00001450*, which resides at an antisense location relative to the *PHYHIPL* gene, a protective effector in glioblastoma multiforme (GBM) with an age-dependent survival rate [28] (Fig. 4d). Interestingly, with RNA-seq data from The Cancer Genome Atlas Glioblastoma Multiforme (TCGA-GBM; GBM: *n* = 165 vs. normal: *n* = 5), we found that *HERV_00001450* significantly reduced in GBM compared with normal samples from both GTEx and TCGA [29] (*P* < 2.2 × 10^−16^, Additional file 1: Fig. S3).

### Genetic regulation of physiological hervRNAs

**Fig. 5:**
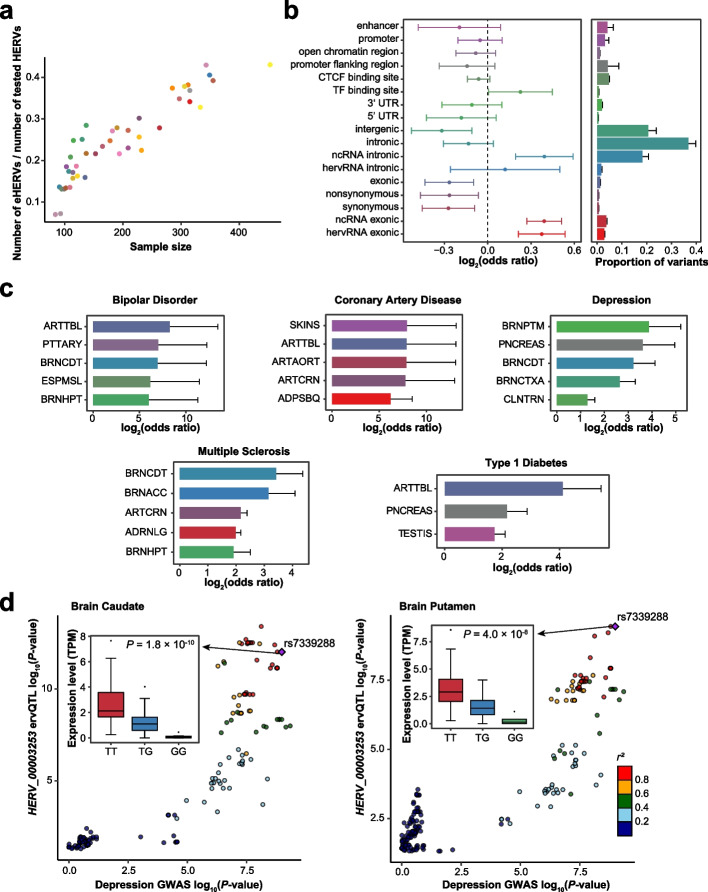
Genetic regulatory effect on hervRNAs. **a** Proportion of eHERVs (*y*-axis) as a function of the sample size for each body site (*x*-axis). See Fig. 2a for the legend of body site-related colors. **b** Functional annotation of *cis*-ervQTLs. Left: *cis*-ervQTL enrichment in functional annotations, shown as the mean ± SD across body sites. Right: proportion of variants across body sites, shown as the mean ± SD. UTR, untranslated region. **c** Top-ranked body sites on the basis of odds ratios of complex disease associations among *cis*-ervQTLs (FDR < 0.05). **d** GWAS colocalization between depression GWAS SNPs and *cis*-ervQTLs of *HERV_00003253* (left: brain caudate, right: brain putamen), generated by LocusCompareR [33]. rs7339288 is the common lead variant with high LD. The boxplots indicate the expression of *HERV_00003253* in different genotypes (from left to right: TT, TG, GG). The center line indicates the median, the limits are the IQR, the whiskers represent 1.5× the IQR, and individual black dots represent outliers

We then examined the effects of genetic variations on hervRNAs by *cis*-ervQTL analysis separately for each body site. Across all body sites, we identified a total of 451,096 *cis*-ervQTLs for 5435 unique eHERVs (HERVs with at least one significantly associated *cis*-ervQTL after permutation and *q*-value correction), which were significantly enriched in full-length HERVs (*P* = 0.011). The numbers of eHERVs varied from 263 in the whole blood to 1359 in the testis (Additional file 2: Table S8). Consistent with previous eQTL studies [30], the eHERV discovery power was found to be positively correlated with sample size (Fig. 5a). To better understand the potential mechanism of genetic regulatory effects, we annotated *cis*-ervQTLs with regulatory annotations of the genome and chromatin state predictions from the Roadmap Epigenomics Project [31] (Additional file 2: Table S9). In contrast to *cis*-eQTLs enriched at canonical splice sites [30], few *cis*-ervQTLs were located at splicing sites (proportion < 0.1%). Instead, *cis*-ervQTLs associated with hervRNA expression levels were most significantly enriched in non-coding RNA-associated regions, suggesting interactions between non-coding RNAs and HERVs [32] (Fig. 5b).

Dysregulation of HERV expression has been implicated in multiple sclerosis, amyotrophic lateral sclerosis, and other complex diseases [34–37]. With GWAS association summary statistics across 22 complex diseases [30, 33] (Additional file 2: Table S10), we identified 19 hervRNA-associated diseases through enrichment analysis (Additional file 1: Fig. S4 and Additional file 2: Table S11). Notably, Parkinson’s disease, schizophrenia, and systemic lupus erythematosus were enriched in almost all solid tissues. Body sites relevant to five diseases, including bipolar disorder, coronary artery disease, depression, multiple sclerosis, and type 1 diabetes, were among the most enriched body sites (Fig. 5c). Through a colocalization analysis including all hervRNA-associated diseases, we detected 102 hervRNAs associated with 15 diseases (Additional file 2: Table S12). After excluding 68 disease-associated hervRNAs for seven autoimmune diseases, we identified 12 hervRNAs from the most biologically relevant body sites for 6 diseases [schizophrenia (*n* = 4), bipolar disorder (*n* = 2), depression (*n* = 1), amyotrophic lateral sclerosis (*n* = 1), atrial fibrillation (*n* = 3), and coronary artery disease (*n* = 2)]. Among these hervRNAs, one is inserted in the disease-associated host gene and half reside at the antisense region of genes with relative biological phenotypes. For example, the depression-related hervRNA *HERV_00003253*, antisense to *B3GLCT* that is involved in synaptogenesis, was only expressed in the brain caudate and brain putamen, which are considered important nuclei for depression [38–40] (Fig. 5d).

### Epigenetic regulation of physiological hervRNAs

**Fig. 6:**
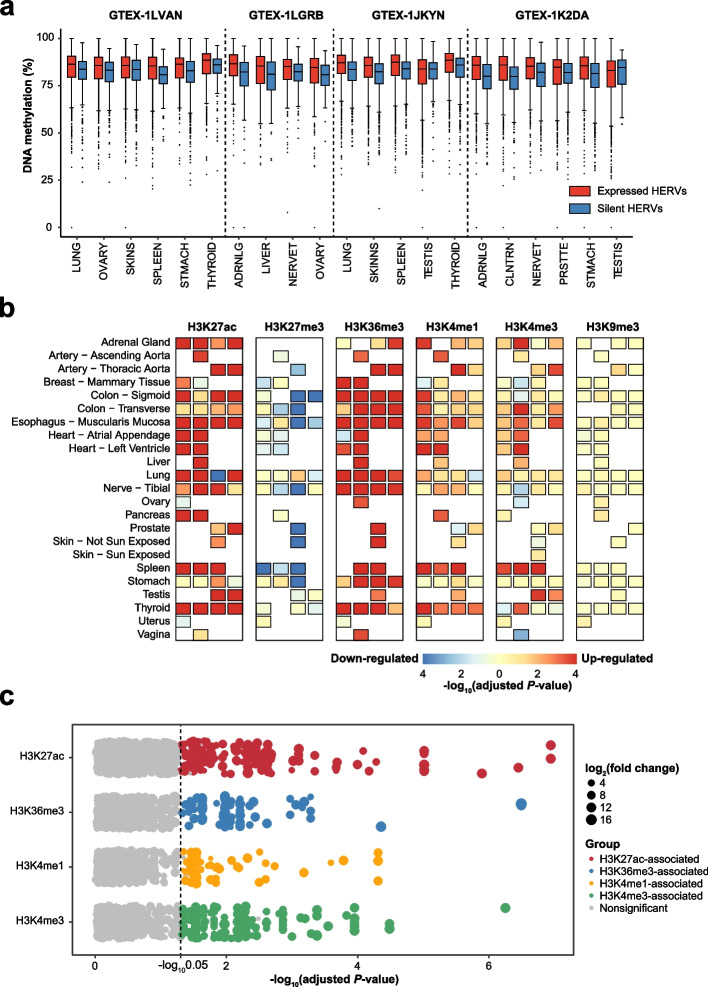
Epigenetic regulation of hervRNAs. **a** The boxplot shows the distribution of the DNA methylation levels of expressed HERVs (TPM ≥ 0.1) and silent HERVs (TPM = 0) at each body site. The difference between the expressed HERVs and silent HERVs at each body site except for the testis was significant (*P* < 0.01, Wilcoxon rank-sum test). The center line indicates the median, the limits are the IQR, whiskers represent 1.5× the IQR, and individual black dots represent outliers. **b** Association between hervRNA expression and histone modification within each body site in each individual (from left to right: GTEX-1LVAN, GTEX-1LGRB, GTEX-1JKYN, and GTEX-1K2DA). **c** Association between the expression of each HERV locus and histone modification across all body sites

Both DNA methylation and histone modification are involved in regulating HERV activity [41, 42]. Thus, we explored the potential epigenetic regulatory mechanism of hervRNAs under physiological conditions by integrating transcriptomic and epigenomic data from 4 individuals included in the ENTEx Project (Additional file 2: Table S13). Although DNA demethylation has been found to contribute to the expressional activation of HERVs, the median DNA methylation level of physiologically expressed HERVs (TPM ≥ 0.1) was over 80%, which was even significantly higher than that of silent HERVs (TPM = 0) in all body sites except the testis, suggesting that DNA demethylation may not be the dominant mechanism for the physiological expression of HERVs (Wilcoxon rank-sum test, FDR < 0.05; Fig. 6a). Then, we performed an enrichment analysis of hervRNAs among six histone modification markers, including H3K27 acetylation (H3K27ac), H3K27 trimethylation (H3K27me3), H3K36 trimethylation (H3K36me3), H3K4 monomethylation (H3K4me1), H3K4 trimethylation (H3K4me3), and H3K9 trimethylation (H3K9me3). At almost all body sites, HERVs located in H3K27ac or H3K36me3 peak regions were significantly more highly expressed (Wilcoxon rank-sum test, FDR < 0.05; Fig. 6b).

For HERV loci analysis, we found that no expressed HERVs were associated with DNA methylation (Additional file 2: Table S14). By contrast, we identified 334 expressed HERVs under the regulation of histone modification (H3K27ac-associated HERVs: *n* = 162; H3K36me3-associated HERVs: *n* = 78; H3K4me1-associated HERVs: *n* = 54; H3K4me3-associated HERVs: *n* = 160; Additional file 2: Table S15). Notably, these expressed HERVs are significantly enriched in chimeric HERVs that concatenate multiple HERV elements (*P* = 4.2 × 10^−5^; Fig. 6c). For example, *HERV_00002430*, a transverse colon-specific expressed HERV that is enriched by H3K27ac signals, concatenate *ERV_645668_LTR12* and *ERV_645667_ERVL-B4-int,MLT2B4*, the families of which were verified to be up-regulated by increased H3K27ac deposition [43].

## Discussion

As one main class of transposable elements from ancient retroviral infections, HERVs are long considered as genomic threats and thus in transcriptional silence regulated by the host [42]. Once aberrantly activated, HERVs may trigger multiple sclerosis, amyotrophic lateral sclerosis, and other complex diseases [4, 34, 35]. However, increasing evidence implied that there may be plenty of expressed HERVs in normal tissues [9, 10, 44, 45]. In our study, we identified more than ten thousand expressed HERV loci across human body sites, especially in the testis and brain. These abundant hervRNAs exhibit body site-specific and biology (sex, ethnicity, and age)-associated expression patterns, which suggest that HERVs are physiologically important rather than constitute “junk DNA.” Although the roles of these hervRNAs, either non-coding hervRNAs or hervRNAs with open reading frames, are largely unknown, our study provides a special RNA “catalog,” which calls for future study on the function of hervRNAs and even potential HERV peptides in physiological or pathological conditions.

Cumulative studies have revealed the activating mechanism of HERVs in complex diseases [34, 35], yet the regulation of HERV expression under physiological conditions remains largely unknown. Our study indicated that physiologically activation of HERVs is under both genetic and epigenetic regulation. By introducing ervQTLs, we identified thousands of hervRNAs regulated by genetic variants, a subset of which were associated with the pathogenesis of multiple complex diseases. In addition, strong enrichment of ervQTLs at non-coding RNA regions suggested the interaction between HERVs and non-coding RNAs. On the other hand, although DNA methylation has been acknowledged to repress HERV activity to maintain genome stability in early developmental stages [46], our study revealed that the expression of HERVs at adult body sites is more likely to be driven by histone modifications, such as H3K27ac and H3K36me3, instead of DNA demethylation. Furthermore, expressed HERVs exhibited even higher DNA methylation, implying negative feedback-regulated DNA methylation under physiological conditions.

Besides broad HERV activity that has been detected in reproductive tissues [47], our study highlights the involvement of hervRNAs in brain development. We found that hervRNAs are abundantly expressed in the brain, at levels second only to those in the testis. On the other hand, our *cis*-ervQTL analysis revealed a strong association between hervRNAs and brain diseases including neurodegenerative and psychiatric diseases. In addition, hervRNAs that show decreased levels with age are also significantly enriched in the brain, especially the hippocampus. As chronic activation of mouse ERV is associated with hippocampus-based memory impairment [48], hervRNAs may also exert functions in human memory and cognition. We also found that hervRNAs are preferentially expressed in the cerebellum, and hervRNAs in the cerebellum are associated with amyotrophic lateral sclerosis, implying that hervRNAs may be involved in cerebellum function. Together, our study suggests that hervRNAs may be involved in brain development as well as cognition. Additionally, the identification of sex-biased and ethnicity-biased hervRNAs in the brain might further our understanding of sex and ethnicity differences in brain structure and function [49, 50].

Although past studies have illustrated the involvement of expressed HERVs in various biological processes and complex diseases [4], most results were based on the family level due to the repetitive nature of HERVs. However, such family-based approaches are unable to decide whether elements within certain HERV families function as general byproducts (such as microRNAs) or in a locus-specific manner [11]. To overcome this difficulty, recent studies have attempted TE-dedicated computational approaches based on RNA sequencing [12, 14, 15, 24, 51]. In the current study, we improved an assembly-based pipeline for locus-specific identification and quantification of expressed HERVs from RNA-seq data. Benefiting from transcript assembly, we are able to decipher the actual transcription of HERV loci and potential regulatory mechanisms. However, due to the individual difference in HERV insertion, mapping these transcripts to the reference genome may not be able to localize their real positions in the genome. In addition, multiple-mapping transcripts of hervRNAs were difficult to be assigned to a specific locus and thus dismissed in the current study. The landscape of hervRNAs would be further locus-specifically drawn with long-read sequencing technologies. Nevertheless, by applying our pipeline to the huge RNA-seq data of the current database, the roles of HERVs will be more comprehensively elucidated in human development and maintenance as well as complex diseases.

## Conclusions

In summary, our work identified and quantified more than ten thousand hervRNAs across normal body sites and focused on the biological insights of hervRNAs in a locus-specific manner. With the resolution of the loci, we revealed that physiologically expressed hervRNAs not only exhibited body site-specific and biology (sex, ethnicity, and age)-associated expression patterns, but also were regulated by genetic variations and histone modifications, suggesting a hidden layer of genetic architecture in human development as well as diseases. The identified hervRNA loci, which may be associated with brain development, cognition, and complex diseases especially neurodegenerative and psychiatric diseases, will lead to novel mechanisms for human development and pathogenesis.

## Methods

### Public dataset description

In total, we collected 9466 samples across 42 body sites from 686 individuals in the GTEx Project [52], including RNA-seq data (FASTQ files), imputation genotyping data (VCF file), and whole-genome sequencing (WGS) data (FASTQ files). The details regarding the samples and sequencing methods are available by dbGaP (study accession: phs000424.v8.p2). From the 1000 Genomes Project [22, 23], we obtained RNA-seq data (FASTQ files) from 372 lymphoblastoid cell line samples through Geuvadis (details in https://www.internationalgenome.org/data-portal/sample). We also downloaded RNA-seq data (FASTQ files) from 165 GBM samples and 5 normal samples from the TCGA-GBM project [29] by dbGaP (study accession: phs000178.v11.p8). In addition, RNA-seq data (FASTQ files) and epigenomic processed data [whole-genome bisulfite sequencing (WGBS) and histone ChIP-seq (H3K27ac, H3K27me3, H3K36me3, H3K4me1, H3K4me3, and H3K9me3)] from four individuals in ENTEx were collected from the ENCODE project [20, 21] (details in https://www.encodeproject.org/entex-matrix/?type=Experiment&status=released&internal_tags=ENTEx).

### Pipeline for the detection of expressed HERVs

First, RNA-seq data were aligned to the reference human genome (GRCh38) using STAR v2.7.5c [53] with Gencode v31 [54] for gene annotation. Reads that mapped to the HERV regions annotated by the HERVd database [55, 56] but did not overlap with transcripts in Gencode were extracted with sambamba v0.6.6 [57] and de novo assembled with Trinity v2.1.1 [58, 59]. The assembled transcripts were quantified using align_and_estimate_abundance.pl in Trinity and remapped to the reference human genome using GMAP version 2020-06-30 [60]. The assembled transcripts with a count > 5 and identity ≥ 96% relative to the reference genome were retained for meta-assembly performed by TACO v0.7.3 [61]. Then, merged HERV candidates identified in ≥ 50% of samples and with a length of ≥ 200 bp were remapped to the reference genome with GMAP. The genes that were uniquely mapped to the HERV loci were retained as candidate HERV genes. Finally, RNA-seq data were realigned to the reference human genome using STAR with the combination of Gencode GTF and the candidate HERV annotations for downstream HERV quantification. HERV expression was calculated at the gene level using RSEM v1.2.28 [62], which is adapted for repeat element quantification. HERVs with a raw count > 5 and TPM ≥ 0.1 [52] among ≥ 50% of samples were considered to be expressed.

### Cell culture and RNA isolation

The HSF cell line (CTCC-003-0165) derived from human normal skin tissue was purchased from the Chinese Tissue Culture Collection (www.ctcc.org). HSF cell line is not listed in the database of commonly misidentified cell lines maintained by ICLAC. It was authenticated not a match for any profile in the DSMZ STR database and tested negative for mycoplasma contamination. HSF was cultured under 37 °C/5% CO_2_ conditions in complete medium (DMEM + 15% FBS). RNA was isolated from cell pellets containing 5 × 10^6^ cells using the FastPure® Cell/Tissue Total RNA Isolation Kit V2 (Vazyme).

### Experimental validation of full-length expressed HERVs

The isolated total RNA was subsequently reverse transcribed into cDNA by using a HiScript® III 1st Strand cDNA Synthesis Kit (+ gDNA wiper) (Vazyme). We designed primers at both ends of the HERV genes (Additional file 2: Table S1) and amplified the cDNAs for 30 cycles with Green Taq Mix (Vazyme). The final PCR products were analyzed by 2% agarose gel electrophoresis and sequenced by Sanger sequencing.

### Quantitative PCR

Transcribed cDNA was quantified using quantitative PCR with ChamQ Universal SYBR qPCR Master Mix (Vazyme). The threshold cycle (Ct) values of the selected HERV genes were normalized to the housekeeping gene *GAPDH*. Relative expression was calculated by adopting the 2^−ΔCt^ method (ΔCt = Ct_HERV_ − Ct_GAPDH_). The primer sequences are shown in Additional file 2: Table S1.

### HERV expression analysis

To remove the redundant HERV genes from different body sites, we clustered them with gffread v0.11.6 [63] (https://github.com/gpertea/gffread) and renamed them with unified gene IDs. Expressed HERVs were annotated with the HERVd database by using BEDTools v2.27.1 [64]. The coding potential of hervRNAs was calculated by CPC2 [65], CPAT v3.0.4 [66], and Pfamscan v1.6 [67] with default parameters. The overlapped outputs from CPC2, CPAT, and Pfamscan were considered as hervRNAs with coding potential.

The similarity of HERV gene expression across samples and between body sites was evaluated using multidimensional scaling with the *cmdscale* function of R v3.6.2. The expression levels were normalized according to a log2-transformed scale [log_2_(TPM + 1)]. The distance between samples was defined as *distance = 1 − correlation* (Spearman). Pairwise differential gene expression analysis was performed using DESeq2 [68] with raw read counts as the input. The differentially expressed HERVs were filtered according to an FDR < 0.05.

### Weighted gene correlation network analysis

We performed a weighted gene correlation network analysis with the *WGCNA* package [69]. The expression levels were normalized using a log2-transformed scale [log_2_(TPM + 1)]. Adjacency matric was calculated using soft thresholding power 6 and turned into a topological overlap matrix (TOM). Then, we did hierarchical clustering with the consensus TOM and identified modules using the Dynamic Tree Cut algorithm. To merge modules with similar expression profiles, we calculated their eigengenes (MEs) and clustered them on their consensus correlation.

### HERV knockdown

The day before transfection, we seeded HSF cell lines in a 6-cm dish to obtain 40–50% confluency at the time of transfection. Cells were transfected with a 10-nM concentration of *HERV_00001917*-target siRNA (5′-GAUGUAAUGAUCAAUGUCCUAUGUC-3′) or non-targeting control siRNA that had been formulated with INTERFERin® transfection reagent (Polyplus) for three biological replicates. As both *HERV_00001917* and target siRNA are unique sequence when aligning to the human genome and transcriptome by BLAST [70], we considered the siRNA as specific to the target hervRNA. Two days post-incubation, RNA was isolated using the FastPure® Cell/Tissue Total RNA Isolation Kit V2 (Vazyme) and sent for RNA sequencing (Paired-end 150 bp) on Illumina NovaSeq 6000.

RNA sequencing reads were aligned to the reference human genome (GRCh38) using STAR with the combination of Gencode GTF and the HERV annotations of HSF in GTEx Project. Gene expression levels were estimated by RSEM and differentially expressed genes were identified by DESeq2. The Gene Ontology enrichment analysis was conducted by the clusterProfiler package [71].

### Evaluation of the effect of sex, ethnicity, and age on hervRNA variation

We performed differential expression analyses related to sex and ethnicity among each body site with DESeq2. Pearson correlations between age and HERV gene expression were calculated for each body site. For differentially expressed HERVs, we analyzed the contribution of sex, ethnicity, or age to hervRNA variation by using a linear mixed model (LMM) implemented in the R package *lme4* [72]. We applied the model as reported in a previous study [25]:

$${H}_{ijk}={B}_i+{I}_j+{C}_k+{E}_{ijk}$$

where *B*_*i*_ denotes the fixed effect of the *i*th body site, *I*_*j*_ denotes the random effect of the *j*th individual, *C*_*k*_ denotes the biological factor effect of the *k*th level, *E*_*ijk*_ represents the random error, and *H*_*ijk*_ represents the *ijk*th HERV expression value at the *i*th body site of the *j*th individual and *k*th level of the factor. Sex and ethnicity were fixed factors, and age was a covariate. Multiple testing was corrected using the Benjamini-Hochberg (BH) method (FDR < 0.05).

### HERV expression of GBM

With the combination of Gencode GTF and the HERV annotations of the brain cortex in the GTEx Project, RNA sequencing reads were aligned to the reference human genome (GRCh38) using STAR. HERV expression levels were estimated by RSEM and normalized by TPM. The comparison of *HERV_00001450* expression levels between GBMs and normal samples from TCGA and GTEx was performed with a two-sided Wilcoxon rank-sum test.

### *cis*-ervQTL analysis

According to the protocol of the GTEx consortium [52], *cis*-ervQTL mapping was performed for all body sites with QTLtools v1.3.1 [73].

As HERV genes exhibit great individual variation, a zero count of a HERV in the quantification analysis might be due to the absence of that HERV gene sequence in an individual genome. Thus, we detected the presence of these HERVs using WGS data. First, we aligned WGS reads to the reference human genome (GRCh38) using BWA v0.7.17 [74]. Target HERV reads were extracted using sambamba and were de novo assembled with ABySS v2.2.3 [75]. Then, we remapped the assembled contigs to the reference genome with BWA. HERVs without unique mapping contigs were considered missing genes, and the expression levels were replaced by missing values (*NA*) for *cis*-ervQTL analysis. HERV expression levels were normalized using TMM [76] and subjected to inverse normal transformation across samples.

To detect hidden batch effects in the HERV expression data, we applied the probabilistic estimation of expression residuals (PEER) method for each body site [77]. The number of PEER factors was in accordance with that in the GTEx study [52]: 15 factors for body sites with fewer than 150 samples, 30 factors for body sites with ≥ 150 and < 250 samples, 45 factors for body sites with ≥ 250 and < 350 samples, and 60 factors for body sites with ≥ 350 samples.

The phased array VCF was filtered according to the following thresholds: missing rate < 5%, minor allele frequency (MAF) < 1%, and Hardy-Weinberg equilibrium (HWE) *P* < 10^−6^. We generated linkage disequilibrium (LD)-pruned variants using PLINK v1.90 [78] with the parameter “--indep-pairwise 200 100 0.2” and calculated the principal components (PCs) of the genotyped variants with EIGENSTRAT [79]. The first three PCs were used as covariates in the *cis*-ervQTL analysis.

We performed *cis*-ervQTL mapping using the nominal and permutation modes (*n* = 10,000) in QTLtools. The *cis* window was defined as ± 1 Mb from the TSS. For each body site, variants in the VCF were further filtered based on minor alleles present in ≥ 10 samples and an MAF ≥ 1%. The expression levels were corrected according to the covariates including genotype PCs, PEER factors, and sex. We calculated *q*-values after permutation and identified eHERVs with at least one significant *cis*-ervQTL (FDR < 0.05). *cis*-ervQTLs with a nominal *P*-value below the threshold calculated by QTLtools were considered significant variants (eVariants) associated with eHERVs.

### Functional enrichment of *cis*-ervQTLs

eVariants were annotated with regulatory annotations for the human genome using ANNOVAR version 2016-02-01 [80] and with 15-state chromatin state predictions from the Roadmap Epigenomics Project with BEDTools. Background variant sets were constructed by matching eVariants to randomly selected variants based on the MAF, chromosome, and distance to the nearest TSS. Due to low counts, the categories ncRNA_splicing, splicing, stopgain, and stoploss were removed. Enrichment analysis was performed with a two-tailed Fisher’s exact test, and all *P*-values were corrected using the BH method.

### GWAS enrichment and colocalization

We collected GWAS association summary statistics across 22 complex diseases, including metabolic diseases, cardiovascular diseases, autoimmune diseases, neurodegenerative diseases, and psychiatric diseases (Additional file 2: Table S10). GWAS SNPs with a *P*-value < 5 × 10^−8^ were extracted. For each GWAS SNP, linked SNPs (*r*^2^ > 0.8) calculated by PLINK were also extracted. Background variant sets were constructed by matching eVariants to randomly selected variants based on the MAF, chromosome, and distance to the nearest TSS. Enrichment analysis was performed with a two-tailed Fisher’s exact test, and all *P*-values were corrected using the BH method.

We applied the *coloc* R package [81] to examine the colocalization between ervQTLs and GWAS results. For each GWAS, we extracted the significant SNPs (*P* < 5 × 10^−8^) with the highest statistical significance among all variants within 1 Mb regions. Then, we extracted eHERVs within 1 Mb from these GWAS SNPs. A gene-based posterior probability of colocalization *PP4* > 0.9 was applied to extract causal SNPs.

### Epigenetic regulation analysis

For epigenomic data from the ENTEx Project, we extracted histone peaks within ± 5 kb of the TSSs of expressed HERVs at all body sites identified by SERVE. HERVs without histone peaks at all body sites were removed. The comparison of expression levels between HERVs with and without histone peaks was performed with a two-sided Wilcoxon rank-sum test. For each HERV, the DNA methylation level was calculated for the region ± 5 kb from the TSS. The comparison of DNA methylation between expressed HERVs (TPM ≥ 0.1) and silent HERVs (TPM = 0) was performed with a two-sided Wilcoxon rank-sum test. Pearson correlations between DNA methylation and HERV gene expression were calculated for each HERV locus. All *P*-values were corrected using the BH method.

## Supplementary Information

Additional file 1: Figure S1-S4. Supplementary figure legends and supplementary figures.

Additional file 2: Table S1-S15. Supplementary tables.

Additional file 3. Peer review history.

## Data Availability

All the data (RNA-seq, WGS, genotype data) from GTEx Project are available by dbGaP (study accession: phs000424.v8.p2) [82]. RNA-seq data from the 1000 Genomes Project were obtained through Geuvadis Project [83]. RNA-seq data from TCGA-GBM were obtained by dbGaP (study accession: phs000178.v11.p8) [84]. RNA-seq data and epigenomic processed data [WGBS and histone ChIP-seq (H3K27ac, H3K27me3, H3K36me3, H3K4me1, H3K4me3, and H3K9me3)] from ENTEx are available by ENCODE Project [85]. HSF in-house RNA-seq data produced by this study have been deposited in SRA (PRJNA776713) [86]. The modified pipeline, together with annotations of hervRNAs produced in this study, is available on GitHub (https://github.com/janky-yz/SERVE) [87] and Zenodo (10.5281/zenodo.6540840) [88].

## Abbreviations

1KGP: 1000 Genomes Project
BH: Benjamini-Hochberg
*cis*-ervQTLs: *cis*-ERV-related quantitative trait loci
eHERV: HERVs with at least one significantly associated *cis*-ervQTL after permutation and *q*-value correction
ENCODE: Encyclopedia of DNA Elements
FDR: False discovery rate
GTEx: Genotype-Tissue Expression
GWAS: Genome-wide association study
H3K27ac: H3K27 acetylation
H3K27me3: H3K27 trimethylation
H3K36me3: H3K36 trimethylation
H3K4me1: H3K4 monomethylation
H3K4me3: H3K4 trimethylation
H3K9me3: H3K9 trimethylation
HERV: Human endogenous retrovirus
HSF: Human skin fibroblast
HWE: Hardy-Weinberg equilibrium
IQR: Interquartile range
LD: Linkage disequilibrium
MAF: Minor allele frequency
PC: Principal component
PEER: Probabilistic estimation of expression residuals
PP4: Posterior probability of colocalization
RT-PCR: Reverse transcription PCR
RT-qPCR: Quantitative reverse transcription PCR
SD: Standard deviation
TAD: Topologically associating domain
TCGA-GBM: The Cancer Genome Atlas Glioblastoma Multiforme
TSS: Transcriptional start site
UTR: Untranslated region
WGBS: Whole-genome bisulfite sequencing
WGS: Whole-genome sequencing
