# Supplementary material for: The landscape of hervRNAs transcribed from human endogenous retroviruses across human body sites
Source: Genome Biol. 2022 Nov 3;23:231. doi: 10.1186/s13059-022-02804-w (PMC9632151; doi:10.1186/s13059-022-02804-w)
Supplement: Supplementary file 1 — Additional file 1: Figure S1-S4. Supplementary figure legends and supplementary figures. [file 13059_2022_2804_MOESM1_ESM.pdf]

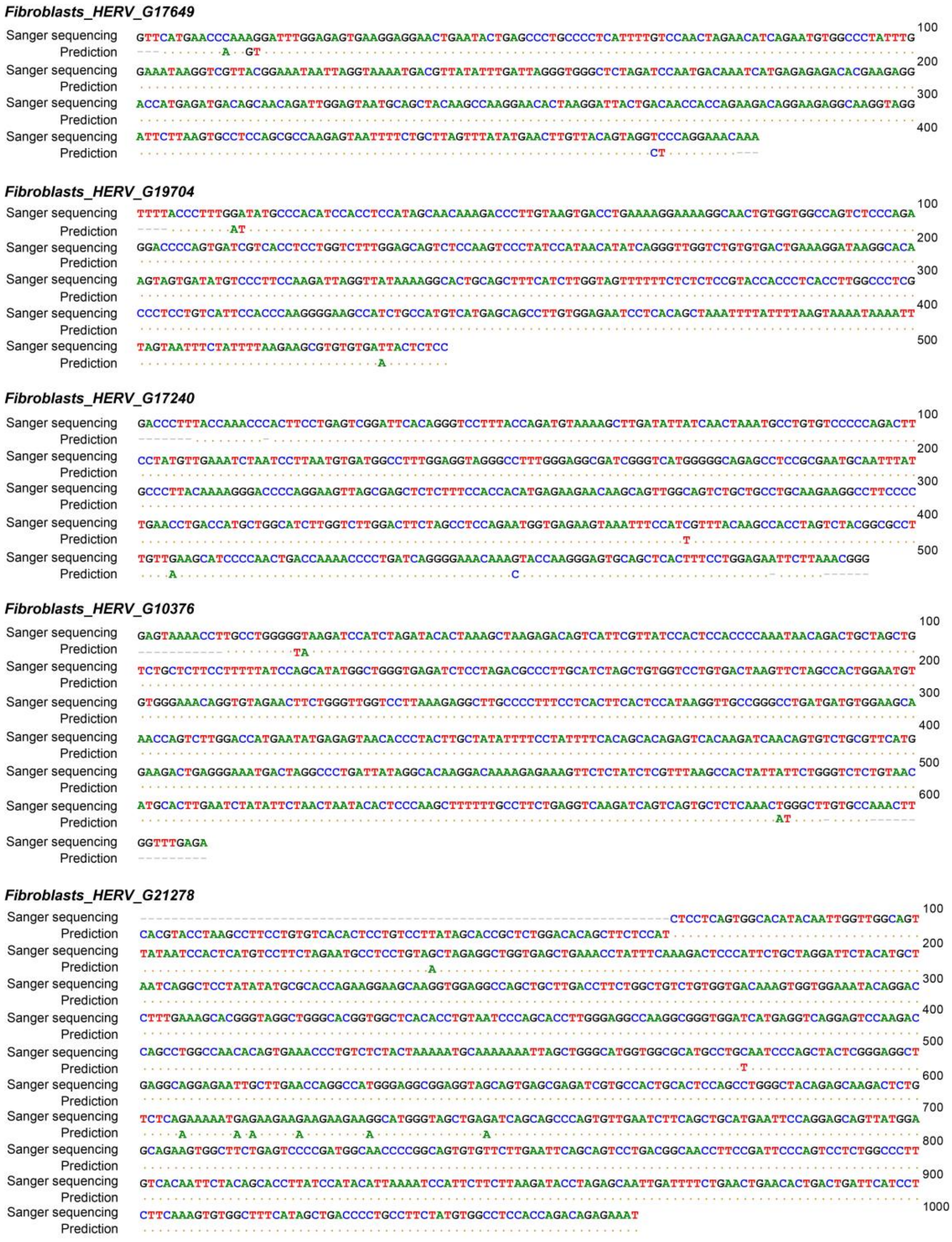

Fig. S1 Sanger sequencing results compared with the prediction of assembly-based pipeline. Expressed HERV sequences predicted by Sanger sequencing and our pipeline. "·" indicates a consistent base and "-" indicates a deletion. The identities of the sequences are all above 98%.

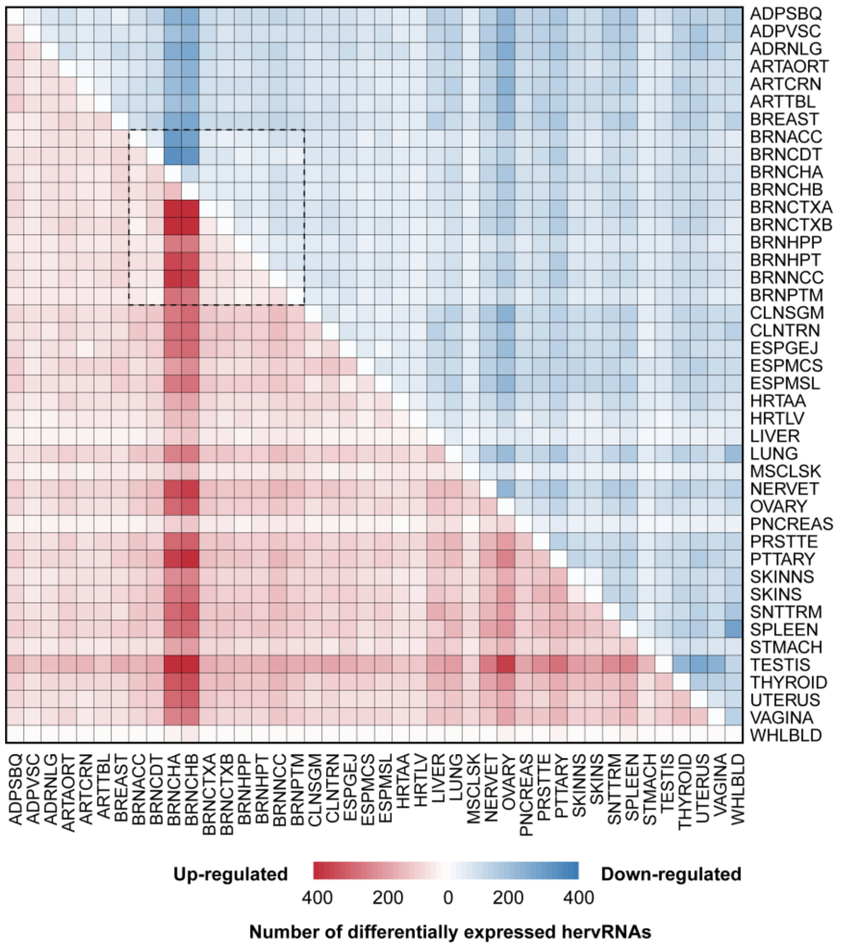

Fig. S2 Pairwise differential expression analysis of hervRNAs between body sites. The differential expression of hervRNAs between body sites was detected with DESeq2. hervRNA expression between brain subregions is shown within the dotted box. The details of the abbreviation are in Additional file2: Table S3.

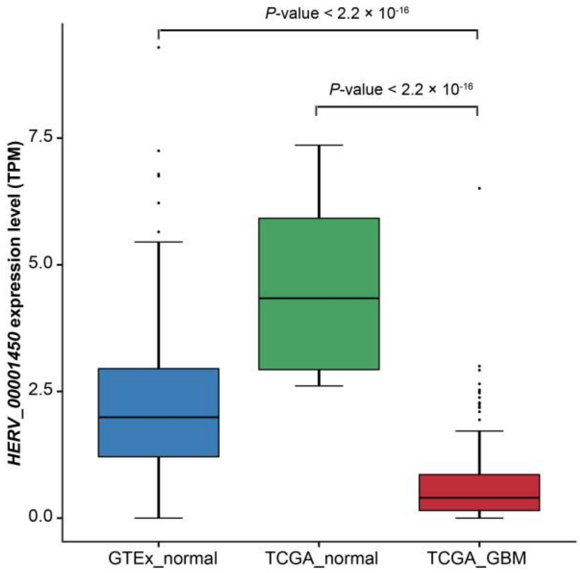

Fig. S3 The association between *HERV_00001450* and GBM. The comparison of *HERV_00001450* expression level between GBM samples (TCGA_GBM) and normal samples (TCGA_normal and GTEx_normal). The center line indicates the median, the limits are the interquartile range (IQR) and the whiskers represent 1.5 × the IQR.

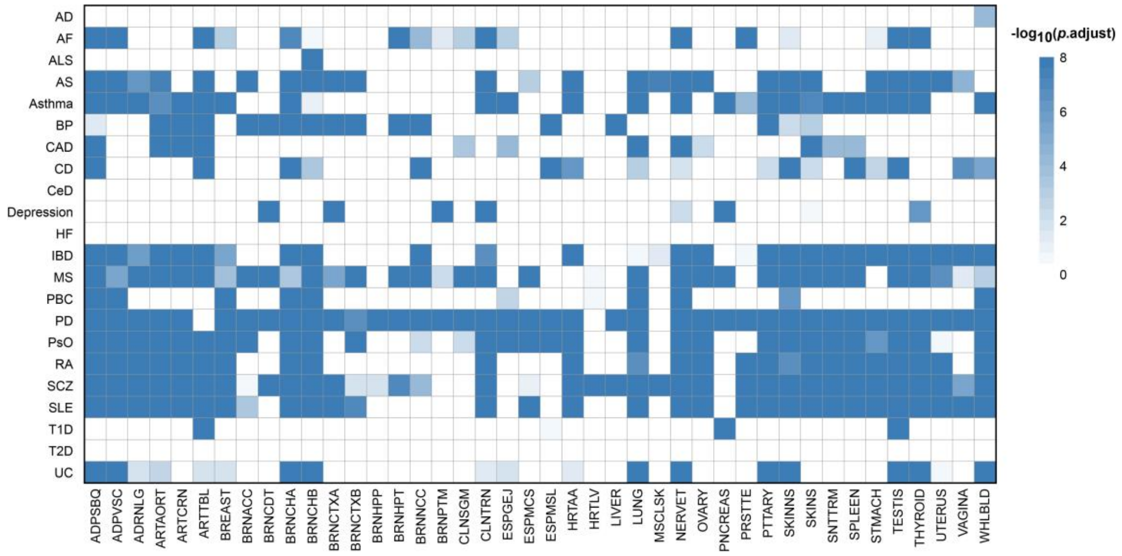

Fig. S4 The association between HERVs and complex diseases. The enrichment of ervQTLs in complex diseases with GWAS association summary statistics across body sites. The details of the abbreviation are in Additional file2: Table S3 (body sites) and S10 (diseases).
