# Supplementary material for: The landscape of hervRNAs transcribed from human endogenous retroviruses across human body sites
Source: Genome Biol. 2022 Nov 3;23:231. doi: 10.1186/s13059-022-02804-w (PMC9632151; doi:10.1186/s13059-022-02804-w)
Supplement: Supplementary file 3 — Additional file 3. Peer review history. [file 13059_2022_2804_MOESM3_ESM.docx]

**Review history**

**First round of review**

**Reviewer 1**

Please consider the following questions:
- Are the methods appropriate to the aims of the study, are they well described, and are necessary controls included? yes
- Are the conclusions adequately supported by the data shown? not all of them Cf. my comments.
- Are sufficient details provided to allow replication and comparison with related analyses that may have been performed? If not, please specify what is required: detils on the sequencing method and conditions.
- Does the work represent a significant advance over previously published studies: Yes
- Is the paper of broad interest to others in the field, or of outstanding interest to a broad audience of biologists: Yes


The manuscript entitled "The landscape of hervRNAs transcribed from human endogenous retroviruses across human body sites" presents a very interesting overview of the major importance of HERVs transcriptional activity in different organs of the human body, with specific trends and signatures between organs but, also between individuals for the same organs.
The global conclusions and the evidenced major transcriptional activity and diversity in the brain are of great interest and add to the existing observations in this domain.
The observation that the epigenetic control is rather exerted by histones, and not much by methylation is also an important observation of the study.
However, as this is about RNA, and because the mode of action, whenever existing like for regulatory RNAs, is unknown, as well as the existence of encoded and actively translated proteins is not studied, the authors cannot evoke "causal roles of RNAs" when detected in diseases. Because of their specific detection, this is of great interest, but one should only speak of "associated RNAs", not causal!
Details on the size of the reads from the RNA sequencing would also be needed, to estimate the rate of inaccurate assemblies of homologous sequences between RNAs from the same HERV family.
Analyzing all these sequences is beyond my possible time spared for it but, having had a glance at randomly selected ones, the following limitations of the study should somehow be discussed:
This is a very intersteing descriptive study with global overview of HERVs transcriptions that already show major "high-level" significant features and specificities, but
-most of these RNAs are non-coding RNAs and heir role(s), if any, has not been studied, but this provides a "catalog" for the purpose that call for future studies on the biological meaning of the numerous HERV RNAs in physiology, and also in diseases.
-RNAs with partial or full-length orfs, would be useful to identify, for future studies on their potential expression as proteins in physiological or pathological conditions.
-However, whatever the % of identity of the reads, the DNA insertion sites of the corresponding RNAs cannot be claimed, all the more when unfixed and/or non-ubiquitous copies are now known to exist, which does not allow to claim the expression from a given loci with certitude by aligning sequences to the reference genomes in the databases. This is where RNAs expressed in certain individuals and not others may be transcribed by non-ubiquitous copies or under different epigenetic regulation, when inserted in different loci between different individuals.

Finally, all the ID, names and references for the RNAs are for molecular (nucleotide) biologists, and do not allow the scientific community studying HERVs with the classical classification of HERV families: HERV+ the letter of amino-acid corresponding to the primer-binding-site anticodon after the R-U5 region, E.g., HERV-K, H, W, E... Even when the R-U5-gag region is not represented, the prototype sequences from each family allow to align the sequences to mots of them. It would be very useful to have the correspondence as far as made possible, to bridge such important transcriptomic studies to the ones dealing with HERV phylogeny or with the identification of HERV-encoded proteins playing a known/shown role in health and/or disease (also depending on when and how expressed).
I wish that this huge "catalog" of sequences may be useful to many future studies by allowing to bridge different disciplines addressing the biology and pathophysiology of HERVs.

**Reviewer 2**

Abstract:
o "HERVs are believed to have lost their transcriptional ability under physiological conditions..." This was very long time ago. Nowadays, everyone on the field knows that it is not true.

o "Combining with genome-wide association study (GWAS), we elucidated that the dysregulation of expressed HERVs might contribute to various complex diseases, especially neurodegenerative and psychiatric diseases." - This conclusion is too far reaching. ERV upregulation has been associated with multiple diseases, however there are only very few causative cases reported. In many cases, the upregulation of ERVs are more considered as a consequence (not a cause) of the disease.


Background / Results:

Lanes 45-46:
"...several expressed HERVs have been found to act as indispensable elements in human development."
These are not simply HERVs, but ERV-derived co-opted/domesticated elements with some novel functionalities. For example, the syncytins are domesticated genes derived from retroviral envelope with a functionality during placentation.

Lanes 48-49:
"Transcriptionally active HERV-H elements are able to affect gene regulatory programs (cite PMID: 25317556), and even create novel topologically associating domains (TADs). [7]"
Not all transcriptionally active HERV-H elements create TADs.

Lanes 56-58:
"However, such family-level approaches are unable to analyze locus-specific regulatory mechanisms and explain whether the observations represent the general
characteristics of all HERV elements within a family or not [7, 10]."
[7] is not a good citation here.

Lanes 103-104:
-"... coding potential was detected in 111 expressed HERVs, including 1 solo-LTR, 28 truncated HERVs, 39 chimeric HERVs and 43 full-length HERVs" - Serious mistake, LTRs are retroviral regulator sequences and do not have coding potential. The given coordinates do not provide a clear ERV.

Lanes 104-108:
It would be important to know which families are providing the different categories.

Lane 106
"...294 of hervRNAs transcribed from upstream..." ? - what the authors mean by upstream? it is not a real definition.

Lanes 111-115:
"Strikingly, nearly four thousand hervRNAs were detected in the cerebellum (Brain - Cerebellum: n = 3,848; Brain - Cerebellar Hemisphere: n = 3,881), which was obviously higher than the numbers at other body sites except for the testis."
Explain, how the normalization was performed.

Lanes 123-132
Body site specific expression: It would be important to know if there was any pattern in the enrichment of a particular HERV family. Provide these information in the cerebellum, testis and liver.

Lanes 131-133
"The high proportion (65.3%) of cerebellum-upregulated and cerebellum-specific hervRNAs implies that hervRNAs present biological functions in cerebellum."
Too far reaching conclusion.

Part discussing ubiquitously expressed hervRNA:
Any pattern regarding ERV family or any other pattern?

Depletion of HERV_00001917-the most highly expressed in the largest module
Knocking down individual sequences of repetitive elements is very challenging. It should be demonstrated that the KD was specific to the targeted site. Knocking out the sequence using CRISPR/Cas9 is the recommended method.

Lanes 157-159:
"...the most sex-biased hervRNA was HERV_00007673, which was brain specific and was significantly highly expressed in males across 6 brain subregions."
What was the most sex-biased hervRNA in the females?

Lanes 174-179:
"HERV_00001450, which resides at an antisense location relative to the PHYHIPL gene, a protective effector in glioblastoma multiforme with an age-dependent survival rate [25]."
Was this particular hervRNA also reduced in the diseased samples of glioblastoma multiforme?

Lanes 178-179:
Again a too far reaching conclusion. On Fig. 4C there are about the similar number of increasing and decreasing hervRNAs - also in the brain. What are those? It is not clear what are the data that suggests that the "...results indicate that hervRNAs may play antiaging roles in the brain."

Genetic regulation from lane 181:
"... eHERVs were significantly enriched in full length HERVs, suggesting that the expression of younger HERVs were more likely to be influenced by genetic variation...".
False conclusion. The full-length ERVs are not necessarily younger in evolution. For example, HERV-Hs are 30MYA (not young) and could have full length copies (more frequently than expected).

Lanes 194-195:
"Instead, cis-ervQTLs in noncoding RNA-associated regions exhibited the strongest effects, suggesting interactions between noncoding RNAs and HERVs."
Not clear at all what the authors mean by this.

Lanes 198-200:
"... Parkinson's disease, schizophrenia, and systemic lupus erythematosus were enriched in almost all solid tissues."
No data are shown, and not obvious to find schizophrenia-associated enrichment in solid tissues...

Lanes 200-212:
No detailed data are shown for this part, and showing HERV_00003253 seems to be a cherry picking.

Lanes 219-222:
" ...the median DNA methylation level of expressed HERVs (TPM ≥ 0.1) was over 80% (median: 83.08%-88.43%), which was significantly 220 higher than that of silent
HERVs (TPM = 0) (median: 79.83%-86.14%; Wilcoxon rank-sum test, FDR < 0.05; Fig. 6a), suggesting that physiologically expressed HERVs are not activated by DNA demethylation." -
Puzzling result with a forced conclusion.

Lanes 253-255:
" ... we identified thousands of hervRNAs regulated by genetic variants, a subset of which contributed to the pathogenesis of multiple complex diseases."
Far reaching, not properly supported conclusion.

"... our study revealed that the expression of HERVs at adult body sites is more likely to be driven by histone modifications, such as H3K27ac and H3K36me3, instead of DNA demethylation."
Was the H3K27me3 demethylation matter opposite to H3K27Ac? Are the samples in the correct order?

Other:
Dissect the different categories from the regulatory aspect:
full-length HERVs, unit-length hervRNAs (intact structure), transcribed from upstream (own regulatory sequences?), 3' readthrough and chimeric (inserted in the transcripts from host genes).

**Authors’ response to reviewers**

Reviewer reports:

Reviewer #1:

Q1.1. Please consider the following questions:
- Are the methods appropriate to the aims of the study, are they well described, and are necessary controls included? yes
- Are the conclusions adequately supported by the data shown? not all of them Cf. my comments.
- Are sufficient details provided to allow replication and comparison with related analyses that may have been performed? If not, please specify what is required: details on the sequencing method and conditions.
We thank for the Reviewer’s reminder and have added details on the sequencing methods and conditions in the Methods (marked as Q1.1).

- Does the work represent a significant advance over previously published studies: Yes
- Is the paper of broad interest to others in the field, or of outstanding interest to a broad audience of biologists: Yes

The manuscript entitled "The landscape of hervRNAs transcribed from human endogenous retroviruses across human body sites" presents a very interesting overview of the major importance of HERVs transcriptional activity in different organs of the human body, with specific trends and signatures between organs but, also between individuals for the same organs.
The global conclusions and the evidenced major transcriptional activity and diversity in the brain are of great interest and add to the existing observations in this domain.
The observation that the epigenetic control is rather exerted by histones, and not much by methylation is also an important observation of the study.

Q1.2. However, as this is about RNA, and because the mode of action, whenever existing like for regulatory RNAs, is unknown, as well as the existence of encoded and actively translated proteins is not studied, the authors cannot evoke "causal roles of RNAs" when detected in diseases. Because of their specific detection, this is of great interest, but one should only speak of "associated RNAs", not causal!
We thank the Reviewer with the comments and revised as suggested (marked as Q1.2).

Q1.3. Details on the size of the reads from the RNA sequencing would also be needed, to estimate the rate of inaccurate assemblies of homologous sequences between RNAs from the same HERV family.
We agree the Reviewer with the professional suggestion. Exactly, the library size (total reads) of the RNA-seq might influence the assemblies of hervRNAs. Thus, in our study, we firstly merged all the samples from a certain body site to produce hervRNA assemblies, which were used for the following quantification of hervRNAs. However, the size of RNA-seq reads of each sample may influence the quantification of hervRNAs, especially for low expressed hervRNAs. So, as suggested, we added details on the size of RNA-seq reads in the supplementary table (Additional file 2: Table S3) and marked as Q1.3.

Q1.4. Analyzing all these sequences is beyond my possible time spared for it but, having had a glance at randomly selected ones, the following limitations of the study should somehow be discussed:
This is a very interesting descriptive study with global overview of HERVs transcriptions that already show major "high-level" significant features and specificities, but
-most of these RNAs are non-coding RNAs and their role(s), if any, has not been studied, but this provides a "catalog" for the purpose that call for future studies on the biological meaning of the numerous HERV RNAs in physiology, and also in diseases.
-RNAs with partial or full-length orfs, would be useful to identify, for future studies on their potential expression as proteins in physiological or pathological conditions.
-However, whatever the % of identity of the reads, the DNA insertion sites of the corresponding RNAs cannot be claimed, all the more when unfixed and/or non-ubiquitous copies are now known to exist, which does not allow to claim the expression from a given loci with certitude by aligning sequences to the reference genomes in the databases. This is where RNAs expressed in certain individuals and not others may be transcribed by non-ubiquitous copies or under different epigenetic regulation, when inserted in different loci between different individuals.
We deeply appreciate these valuable suggestions from the Reviewer. It is to our surprise that there are so many physiologically expressed hervRNAs, which raises up the question about their roles or functions in physiology or diseases. Specially, we are also interested in the coding potential of hervRNAs, and will try to detect potential peptides in the future. In the revised manuscript, we have called for attentions to the function of hervRNAs and marked as Q1.4.1.
We also agree that localization of hervRNAs is a great challenge, due to the individual difference of HERV insertion and expression. In our work, we employed an alternative strategy by mapping assembled transcripts to the reference genome, although the results could not fully localize hervRNAs in individual genomes. We have discussed the limitation of hervRNA localization and marked as Q1.4.2.

Q1.5. Finally, all the ID, names and references for the RNAs are for molecular (nucleotide) biologists, and do not allow the scientific community studying HERVs with the classical classification of HERV families: HERV+ the letter of amino-acid corresponding to the primer-binding-site anticodon after the R-U5 region, E.g., HERV-K, H, W, E... Even when the R-U5-gag region is not represented, the prototype sequences from each family allow to align the sequences to most of them. It would be very useful to have the correspondence as far as made possible, to bridge such important transcriptomic studies to the ones dealing with HERV phylogeny or with the identification of HERV-encoded proteins playing a known/shown role in health and/or disease (also depending on when and how expressed).
I wish that this huge "catalog" of sequences may be useful to many future studies by allowing to bridge different disciplines addressing the biology and pathophysiology of HERVs.
We thank for the Reviewer’s suggestion from the side of experimental biologists. In the revised manuscript, we extracted the HERV families for each hervRNAs from HERVd database, and added the family information in “Additional file 2: Table S2” (marked as Q1.5).


Reviewer #2:

Please consider the following questions:
- Are the methods appropriate to the aims of the study, are they well described, and are necessary controls included? If not, please specify what is required.

The analysis is appropriate

- Are the conclusions adequately supported by the data shown? If not, please explain

In general, several conclusions that might be supported by the data are missing, whereas the conclusions made by the authors are too far reaching and not properly supported

- Are sufficient details provided to allow replication and comparison with related analyses that may have been performed? If not, please specify what is required.

At some parts there are no sufficient details are provided.

- Does the work represent a significant advance over previously published studies?
The work has some merit, but only the pros are discussed and the limitations are not.

- Is the paper of broad interest to others in the field, or of outstanding interest to a broad audience of biologists?


Abstract:
Q2.1. "HERVs are believed to have lost their transcriptional ability under physiological conditions..." This was very long time ago. Nowadays, everyone on the field knows that it is not true.
We thank for the suggestion and revised with straightforward statement as “Human endogenous retroviruses (HERVs), the remnants of ancient retroviruses, account for 8% of human genome, but most have lost transcriptional abilities under physiological conditions” (marked as Q2.1).

Q2.2. "Combining with genome-wide association study (GWAS), we elucidated that the dysregulation of expressed HERVs might contribute to various complex diseases, especially neurodegenerative and psychiatric diseases." - This conclusion is too far reaching. ERV upregulation has been associated with multiple diseases, however there are only very few causative cases reported. In many cases, the upregulation of ERVs are more considered as a consequence (not a cause) of the disease.
We agree with the comment of the Reviewer and toned down the phrase by replacing “contribute to” with “be associated with” (marked as Q2.2).

Background / Results:

Q2.3. Lanes 45-46:
"...several expressed HERVs have been found to act as indispensable elements in human development."
These are not simply HERVs, but ERV-derived co-opted/domesticated elements with some novel functionalities. For example, the syncytins are domesticated genes derived from retroviral envelope with a functionality during placentation.
We agree with the Reviewer’s professional opinion and revised as suggested (marked as Q2.3).

Q2.4. Lanes 48-49:
"Transcriptionally active HERV-H elements are able to affect gene regulatory programs (cite PMID: 25317556), and even create novel topologically associating domains (TADs). [7]"
Not all transcriptionally active HERV-H elements create TADs.
We thank the Reviewer and revised as suggested (marked as Q2.4).

Q2.5. Lanes 56-58:
"However, such family-level approaches are unable to analyze locus-specific regulatory mechanisms and explain whether the observations represent the general characteristics of all HERV elements within a family or not [7, 10]."
[7] is not a good citation here.
Revised as suggested and marked as Q2.5.

Q2.6. Lanes 103-104:
-"... coding potential was detected in 111 expressed HERVs, including 1 solo-LTR, 28 truncated HERVs, 39 chimeric HERVs and 43 full-length HERVs" - Serious mistake, LTRs are retroviral regulator sequences and do not have coding potential. The given coordinates do not provide a clear ERV.
We appreciate the Reviewer with the professional comment. We revised the conclusion as suggested and supplied the details of HERV elements in the supplementary table (Additional file 2: Table S2). A detail revision is marked as Q2.6 in the track-change version of revised MS.

Q2.7. Lanes 104-108:
It would be important to know which families are providing the different categories.
We agree with the Reviewer and added the family information in “Additional file 2: Table S2” (marked as Q2.7).

Q2.8. Lane 106
"...294 of hervRNAs transcribed from upstream..." ? - what the authors mean by upstream? it is not a real definition.
We are sorry to confuse the Reviewer with the classification. Based on a recent review (PMID 32576954), we classified hervRNAs by comparing hervRNA transcripts to HERV elements recording in HERVd database. Thus, hervRNAs transcribed from upstream mean that the transcription start sites of hervRNAs are located at the upstream of HERV elements. We added the definition and citation in the revised MS and marked as Q2.8.

Q2.9. Lanes 111-115:
"Strikingly, nearly four thousand hervRNAs were detected in the cerebellum (Brain - Cerebellum: n = 3,848; Brain - Cerebellar Hemisphere: n = 3,881), which was obviously higher than the numbers at other body sites except for the testis."
Explain, how the normalization was performed.
We are sorry for confusing the Reviewer. Actually, in this result, we just simply described the total number of hervRNAs in each body site without any normalization (Figure 3a). To be clearer, we supplied the amount of hervRNAs in the testis and other body sites in the sentence, showing that the top 3 body sites are Testis, Brain – Cerebellar Hemisphere and Brain – Cerebellum. To avoid misunderstanding, we also deleted the word “obviously”. A detail revision is marked as Q2.9 in the track-change version of revised MS.

Q2.10. Lanes 123-132
Body site specific expression: It would be important to know if there was any pattern in the enrichment of a particular HERV family. Provide these information in the cerebellum, testis and liver.
We further explored the body-site-specific hervRNAs and found that testis-specific hervRNAs were significantly enriched in LTR12C family, which was highly expressed during early spermatogenic stages (PMID: 30315278). For other body sites including cerebellum and liver, we didn’t detect any pattern in the enrichment of a particular HERV family. A detail revision is marked as Q2.10 in the track-change version of revised MS.

Q2.11. Lanes 131-133
"The high proportion (65.3%) of cerebellum-upregulated and cerebellum-specific hervRNAs implies that hervRNAs present biological functions in cerebellum."
Too far reaching conclusion.
We modified our statement and moved into the Discussion section (marked as Q2.11).

Q2.12. Part discussing ubiquitously expressed hervRNA:
Any pattern regarding ERV family or any other pattern?
We appreciate the Reviewer with the inspiring question. However, we failed to get any positive clues about HERV family after various attempts. Given that there are only 127 ubiquitously expressed hervRNAs, it is not very surprising that no HERV families are enriched.

Q2.13. Depletion of HERV_00001917-the most highly expressed in the largest module
Knocking down individual sequences of repetitive elements is very challenging. It should be demonstrated that the KD was specific to the targeted site. Knocking out the sequence using CRISPR/Cas9 is the recommended method.
We fully agree with the Reviewer’s comments. Locus-specific manipulation of HERV loci is a great challenge. However, each hervRNA identified in the current study contains a unique region, due to our assembly-based strategy. The designed siRNA is targeted to the unique region of HERV_00001917. When aligning the siRNA to human reference genome with BLAST, there is also only one hit (see Figure Q2.13.1 in “point-by-point response_GB.docx”). As the expression level of HERV_00001917 were decreased in KD (verified by both RT-qPCR and RNA-seq), and differentially expressed genes did not include other hervRNAs within the same HERV family of HERV_00001917, we thought that the siRNA was specific to the targeted site. We added the explanation in the Methods and marked as Q2.13.
We also agree that KO is a better choice to KD. However, the HSF cell line used in our work is a primary cell line. In addition, hervRNAs are a subclass of lncRNAs, and thus require KO with a pair of gRNAs. These factors make it difficult to establish stable KO clones. That’s why we selected KD in our previous version. With the suggestion, we also tried to knock out HERV_00001917 using CRISPR/Cas9 (Figure Q2.13.2a in “point-by-point response_GB.docx”) and found a subset of HSFs to be successfully KO (Figure Q2.13.2b in “point-by-point response_GB.docx”). However, the cell viability had largely decreased before we further isolated the HERV_00001917-KO clones. Then, we turned to the HSF cell pools but the knockout efficiency was too low for downstream analysis (Figure Q2.13.2c in “point-by-point response_GB.docx”). Thus, we finally failed to achieve KO on HSFs during the revision.

Q2.14. Lanes 157-159:
"...the most sex-biased hervRNA was HERV_00007673, which was brain specific and was significantly highly expressed in males across 6 brain subregions."
What was the most sex-biased hervRNA in the females?
The most sex-biased hervRNA in the females is HERV_00001037, which was most biased in breast across body sites. We added it in the revised MS and marked as Q2.14.

Q2.15. Lanes 174-179:
"HERV_00001450, which resides at an antisense location relative to the PHYHIPL gene, a protective effector in glioblastoma multiforme with an age-dependent survival rate [25]."
Was this particular hervRNA also reduced in the diseased samples of glioblastoma multiforme?
The Reviewer raised up an interesting question that we are also curious about. We further introduced RNA-seq data from TCGA-GBM project (glioblastoma multiforme: n = 165 vs. normal: n = 5) and quantified HERV expression with annotation from brain cortex. Compared with normal samples from GTEx and TCGA database, the expression of HERV_00001450 is significantly reduced (P < 2.2 × 10-16). A detail revision is marked as Q2.15 in the track-change version of revised MS.

Q2.16. Lanes 178-179:
Again a too far reaching conclusion. On Fig. 4C there are about the similar number of increasing and decreasing hervRNAs - also in the brain. What are those? It is not clear what are the data that suggests that the "...results indicate that hervRNAs may play antiaging roles in the brain."
We thank the Reviewer with the comment and agree that the original figure couldn’t provide comprehensive information about the age-associated hervRNAs. Thus, we reorganized the figure with the distribution of both age-associated increasing and decreasing hervRNAs across body sites (Fig. 4c, also in “point-by-point response_GB.docx” for convenience). Although the total number of increasing and decreasing hervRNAs are similar, we found that that age-associated decreasing hervRNAs are enriched in 13 body sites including six brain subregions (Fig. 4c, body sites in blue). Specially, brain hippocampus showed the strongest enrichment of the age-associated decreasing hervRNAs (113/119), suggesting that these hervRNAs might be associated with memory and cognition. For clearer demonstration, we deleted the original conclusion and discussed it in the Discussion section. A detail revision is marked as Q2.16 in the track-change version of revised MS.

Q2.17. Genetic regulation from lane 181:
"... eHERVs were significantly enriched in full length HERVs, suggesting that the expression of younger HERVs were more likely to be influenced by genetic variation...".
False conclusion. The full-length ERVs are not necessarily younger in evolution. For example, HERV-Hs are 30MYA (not young) and could have full length copies (more frequently than expected).
We thanked the Reviewer with the professional comment. We rechecked our data on eHERVs and admitted that this is a false conclusion. So we deleted the sentence in the revised MS and marked as Q2.17.

Q2.18. Lanes 194-195:
"Instead, cis-ervQTLs in noncoding RNA-associated regions exhibited the strongest effects, suggesting interactions between noncoding RNAs and HERVs."
Not clear at all what the authors mean by this.
We are sorry to confuse the Reviewer. In this study, we applied eQTL approaches to detect SNPs associated with hervRNA expression, which would provide clues for dissecting the potential regulatory mechanism of ERV-derived elements. Thus, we found that cis-ervQTL SNPs were significantly enriched in noncoding-RNA-associated regions, i.e., the SNPs of noncoding-RNA-associated regions are associated with hervRNA expression levels, which suggests the interactions between noncoding RNAs and HERVs. For clearer demonstrations, we revised the statements and marked as Q2.18.

Q2.19. Lanes 198-200:
"... Parkinson's disease, schizophrenia, and systemic lupus erythematosus were enriched in almost all solid tissues."
No data are shown, and not obvious to find schizophrenia-associated enrichment in solid tissues...
We are sorry for not showing the supporting data. In the revised MS, we added a supplementary table (Additional file 2: Table S11) and a supplementary figure (Additional file1: Fig. S4, also in “point-by-point response_GB.docx” for convenience) to show the results of disease enrichment, which is marked as Q2.19.

Q2.20. Lanes 200-212:
No detailed data are shown for this part, and showing HERV_00003253 seems to be a cherry picking.
We are sorry to confuse the Reviewer and added a supplementary table (Additional file 2: Table S12) to show the colocalization result of cis-ervQTLs and GWAS (marked as Q2.20).

Q2.21. Lanes 219-222:
" ...the median DNA methylation level of expressed HERVs (TPM ≥ 0.1) was over 80% (median: 83.08%-88.43%), which was significantly higher than that of silent
HERVs (TPM = 0) (median: 79.83%-86.14%; Wilcoxon rank-sum test, FDR < 0.05; Fig. 6a), suggesting that physiologically expressed HERVs are not activated by DNA demethylation." -
Puzzling result with a forced conclusion.
Sorry for confusing. Actually, cumulative studies have revealed that DNA demethylation contribute to abnormal activation of expressed HERVs. However, we found that the median DNA methylation level of physiologically expressed HERVs (TPM ≥ 0.1) was over 80% across body sites, which was even significantly higher than that of silent HERVs (TPM = 0) in all body sites except testis, indicating that DNA demethylation should not be the dominant mechanism for the physiological expression of HERVs. A detail revision is marked as Q2.21 in the track-change version of revised MS.

Q2.22. Lanes 253-255:
" ... we identified thousands of hervRNAs regulated by genetic variants, a subset of which contributed to the pathogenesis of multiple complex diseases."
Far reaching, not properly supported conclusion.
We are sorry for not providing the supporting data and supplied all data including two supplementary tables and a supplementary figure, which are marked as Q2.19 and Q2.20. The conclusion was also revised and marked as Q2.22.

Q2.23. "... our study revealed that the expression of HERVs at adult body sites is more likely to be driven by histone modifications, such as H3K27ac and H3K36me3, instead of DNA demethylation."
Was the H3K27me3 demethylation matter opposite to H3K27Ac? Are the samples in the correct order?
We thank the Reviewer proposed a question that we were also curious. First, the samples are in the correct order, but not all the samples were sequenced with all the histone modifications. For the question about the relationship of H3K27ac and H3K27me3, the current data indicated no opposite relationship between them (Fig 6b). In details, HERVs located in H3K27ac peak regions were significantly more highly expressed at almost all body sites in all individuals. By contrast, HERVs located in H3K27me3 peak regions were found significantly more lowly expressed at most body sites in only one individual. So we can’t guarantee the association of physiologically expressed HERVs and H3K27me3, which may due to the individual difference and the limited size of samples. Thus, we did not modify manuscript about this point.

Q2.24. Other:
Dissect the different categories from the regulatory aspect:
full-length HERVs, unit-length hervRNAs (intact structure), transcribed from upstream (own regulatory sequences?), 3' readthrough and chimeric (inserted in the transcripts from host genes).
We appreciated the Reviewer for the professional suggestion. Actually, although we have revealed that HERVs under genetic regulation were enriched in full-length HERVs, we found no significant enrichment for other categories. Among disease-associated HERVs and HERVs under epigenetic regulation, the number of full-length HERVs are 6 and 12, which are not enough for further dissection. Thus, we did not modify manuscript for this question, either.
